# The pulmonary artery catheter in modern anesthesiology and intensive care: indications, benefits, and limitations

**DOI:** 10.1016/j.bjane.2025.844587

**Published:** 2025-01-14

**Authors:** Andre P. Schmidt, Clovis T. Bevilacqua Filho, Eduarda S. Martinelli, Virgínia C. de Moura

**Affiliations:** a Hospital de Clínicas de Porto Alegre (HCPA), Serviço de Anestesia e Medicina Perioperatória, Porto Alegre, RS, Brazil; b Santa Casa de Porto Alegre, Serviço de Anestesia, Porto Alegre, RS, Brazil; c Hospital Nossa Senhora da Conceição, Serviço de Anestesia, Porto Alegre, RS, Brazil; d Universidade Federal do Rio Grande do Sul (UFRGS), Programa de Pós-Graduação em Ciências Pneumológicas, Porto Alegre, RS, Brazil; e Universidade Federal do Rio Grande do Sul (UFRGS), Programa de Pós-Graduação em Ciências Cirúrgicas, Porto Alegre, RS, Brazil; f Faculdade de Medicina da Universidade de São Paulo (FMUSP), Programa de Pós-Graduação em Anestesiologia, Ciências Cirúrgicas e Medicina Perioperatória, São Paulo, SP, Brazil; g University Health Network, Toronto General Hospital, Department of Anesthesia and Pain Management, Toronto, Canada; h University of Toronto, Temerty Faculty of Medicine, Department of Anesthesiology and Pain Medicine, Toronto, Canada

The pulmonary artery catheter (PAC), also known as the Swan-Ganz catheter, has been a cornerstone of hemodynamic monitoring since its introduction in the 1970s.^1^ Over the decades, the utility of the PAC has been extensively debated in critical care and perioperative medicine, leading to a reevaluation of its indications and a redefinition of its role in patient management. Despite the controversies, the PAC remains an invaluable tool for specific clinical scenarios. This editorial examines the current status of PACs, with emphasis on their indications, benefits, pitfalls, and the nuanced decisions required for their effective use in intensive care and surgical settings.

The PAC was initially developed to measure hemodynamic parameters in patients with acute myocardial infarction and heart failure.^2^ Over time, its application expanded to critically ill patients and perioperative monitoring in high-risk surgical procedures. The catheter provides real-time measurements of pulmonary artery pressures, cardiac output, mixed venous oxygen saturation, and pulmonary vascular resistance, offering a comprehensive hemodynamic profile.^3^ Today, PACs are predominantly used in the following scenarios: diagnosis and management of severe cardiogenic shock, where they facilitate comprehensive hemodynamic assessment; suspected or known pulmonary artery hypertension; the evaluation and management of patients with conditions such as unexplained or unknown volume status in shock; determine candidacy for mechanical circulatory support; or severe underlying cardiopulmonary disease (e.g., congenital heart disease, left-to-right shunt, severe valvular disease, or pulmonary hypertension) undergoing corrective or other surgery.^4^, ^5^, ^6^ Right heart catheterization remains the gold standard for diagnosing pulmonary hypertension and assessing advanced heart failure, especially in the context of heart replacement therapies.^7^^,^^8^ Intraoperative use of PACs during cardiac and high-risk non-cardiac surgeries enables detailed monitoring of right ventricular function and fluid management, particularly in patients with complex cardiovascular profiles.^9^^,^^10^ When less invasive methods fail to elucidate the cause of shock, PACs provide invaluable data to tailor therapy.^11^

The PAC's ability to measure pressures and cardiac output directly ensures precise diagnosis of hemodynamic derangements, including preload, afterload, and contractility abnormalities. This is especially useful in distinguishing between cardiogenic and distributive shock.^12^ PAC-guided therapies have been associated with improved outcomes in certain populations. Early insertion and comprehensive hemodynamic profiling in cardiogenic shock reduce mortality compared to delayed or absent PAC use.^6^^,^^13^ Moreover, PAC data guide the titration of inotropes, vasopressors, and diuretics, optimizing patient management. Intraoperative PAC use in cardiac surgery has shown benefits in monitoring and managing right ventricular dysfunction, often underrecognized with standard monitoring techniques.^10^ Advanced hemodynamic parameters derived from PACs, such as the coupling ratio and myocardial reserve, are increasingly recognized for their prognostic significance in advanced heart failure and shock.^14^

Despite its advantages, PAC use is not without challenges and pitfalls. The routine use of PACs in surgical and critically ill patients has faced considerable scrutiny over the past decades. Large observational studies, small randomized trials, and meta-analyses have consistently demonstrated no improvement in survival or reductions in hospital stays with the routine use of PAC in these populations.^15^, ^16^, ^17^, ^18^ A meta-analysis by Shah et al.^19^ identified no significant improvement in overall survival among critically ill patients managed with PACs, raising questions about its clinical utility. Similarly, the Evaluation Study of Congestive Heart Failure and Pulmonary Artery Catheterization Effectiveness (ESCAPE) trial demonstrated no mortality benefit with PAC use in patients with shock.^20^ Complications such as infections, thrombosis, and pulmonary artery rupture also contribute to the risk profile, as evidenced by Rong et al.,^9^ who noted increased in-hospital mortality in cardiac surgery patients using PACs. Accurate interpretation of PAC-derived data requires significant expertise. Misinterpretation can lead to inappropriate interventions, worsening patient outcomes.^21^ Compared to methods or devices like echocardiography, PACs are costly and invasive, necessitating trained personnel and increasing procedural complexity.^22^ For critically ill patients without underlying cardiac disease, the benefits of PAC monitoring are less clear, and less invasive modalities may suffice.^23^ These findings have led to a substantial decline in the use of PACs, with a U.S. study reporting a 65% reduction in their utilization between 1993 and 2004.^24^ Consequently, most societal guidelines have recommended against the routine use of PACs, even in higher-risk populations undergoing noncardiac surgery.^25^^,^^26^ These limitations underscore the need for careful consideration when deciding on PAC use in clinical practice.

More recently, the PAC has undergone a resurgence in specific contexts, driven by advancements in catheter technology and refined patient selection criteria. Recent efforts aim to address its limitations while emphasizing its strengths. Despite the lack of demonstrated survival benefit, there remains a nuanced role for PACs in the management of select patient populations. Clinicians experienced in the care of critically ill patients recognize the valuable hemodynamic insights PACs provide in specific scenarios. For example, PACs may be appropriate in cases of complex circulatory or respiratory failure, pulmonary hypertension, or left heart dysfunction, particularly when less invasive methods cannot adequately clarify the hemodynamic status. Modern guidelines recommend PAC use in patients with high-risk cardiovascular profiles, hemodynamic instability, or refractory shock. Improved understanding of phenotypes and staging systems for cardiogenic shock aids in identifying appropriate candidates.^27^ Advances in PAC design, such as third-generation catheters with integrated pulse wave analysis and automated thermodilution, enhance accuracy and expand utility.^3^ Combining PAC data with echocardiographic findings provides a holistic hemodynamic assessment, particularly in ICU settings.^28^ Initiatives to standardize right heart catheterization protocols and improve clinician training aim to reduce complications and improve data interpretation.^14^ High-quality randomized controlled trials are urgently needed to evaluate the efficacy of PAC-guided management in diverse patient populations, particularly in the perioperative and critical care settings.

The key to effective PAC use may lie in its selective application. The decision to place a PAC should be driven by a targeted clinical question regarding a patient's hemodynamic condition that cannot be answered satisfactorily through clinical evaluation or less invasive techniques. If the resulting data could alter management decisions, PAC placement may be justified. Clinicians must also possess the expertise to ensure proper catheter placement, accurate data collection, and reliable interpretation of the results to maximize the benefits while minimizing potential complications. In non-critically ill patients, PACs are less frequently used but remain a valuable tool in evaluating individuals with suspected or confirmed pulmonary hypertension. This underscores the importance of tailoring PAC use to the patient's clinical context and needs, rather than relying on a one-size-fits-all approach. Overall, while the routine use of PACs is no longer supported, their selective use in appropriately chosen scenarios continues to offer significant benefits. The evolving role of PACs highlights the necessity for clinicians to balance evidence-based practice with individualized patient care, ensuring that the use of this tool remains judicious and impactful.

In summary, the PAC remains a relevant tool in the management of critically ill and surgical patients, particularly those with complex hemodynamic derangements. However, its use should be carefully tailored to individual patient needs, balancing potential benefits against risks. Technological advancements, combined with rigorous training and adherence to evidence-based guidelines, can further enhance the utility of PACs. While noninvasive or minimally invasive techniques are valuable alternatives in many scenarios, the PAC's ability to provide direct and comprehensive hemodynamic data ensures its continued relevance in modern anesthesiology and intensive care medicine. The challenge lies in optimizing its application to maximize patient outcomes while minimizing complications and costs.

## Conflicts of interest

The authors declare no conflicts of interest.
